# Draft Genome Sequence of *Aeromonas hydrophila* Strain Ae34, Isolated from a Septicemic and Moribund Koi Carp (*Cyprinus carpio koi*), a Freshwater Aquarium Fish

**DOI:** 10.1128/genomeA.00572-14

**Published:** 2014-06-12

**Authors:** S. S. S. De S. Jagoda, Engkong Tan, Appudurai Arulkanthan, Shigeharu Kinoshita, Shugo Watabe, Shuichi Asakawa

**Affiliations:** a Laboratory of Aquatic Molecular Biology and Biotechnology, Department of Aquatic Bioscience, Graduate School of Agricultural and Life Sciences, the University of Tokyo, Tokyo, Japan; b Center for Aquatic Animal Disease Diagnosis and Research, Department of Veterinary Pathobiology, Faculty of Veterinary Medicine and Animal Science, University of Peradeniya, Peradeniya, Sri Lanka; c School of Marine Biosciences, Kitasato University, Sagamihara, Kanagawa, Japan

## Abstract

*Aeromonas hydrophila* is an important opportunistic pathogen that infects a variety of aquatic and terrestrial animals, including humans. We report here the draft genome sequence of *A. hydrophila* Ae34, a multidrug-resistant isolate from the kidney of a moribund koi carp (*Ciprinus carpio koi*) with signs of hemorrhagic septicemia.

## GENOME ANNOUNCEMENT

*Aeromonas hydrophila* has long been recognized as an opportunistic pathogen causing septicemia in many species of freshwater fish (1). Aquarium-raised tropical ornamental fish are highly prone to infections caused by *A. hydrophila* and other mesophilic aeromonads (2–4), due to various stressors that accompany intensive management practices in commercial ornamental fish production, as well as to the ubiquitous nature of aeromonads in tropical waters. The mortalities associated with these bacterial infections, along with costs related to therapeutic intervention and control measures, cause significant economic losses to the aquarium industry. Moreover, *A. hydrophila* is considered an emerging pathogen in humans (5). Thus, the presence of multidrug-resistant *A. hydrophila* in pet fish and carriage water has numerous public health implications (6). The complete genome sequence of the channel catfish epidemic isolate *A. hydrophila* ML09-119 (7) and the draft genome sequences of some fish-borne *A. hydrophila* clinical isolates (8, 9) were reported recently. However, there is a dearth of information on the genome sequence of *A. hydrophila* isolated from tropical ornamental fish, despite the growing interest and economic importance of the global aquarium fish trade.

*A. hydrophila* strain Ae34 was isolated from the kidney of a moribund septicemic koi carp from a commercial aquarium. Genomic DNA was extracted by using a DNeasy blood and tissue kit (Qiagen). Genome sequencing was performed using the Ion Torrent PGM Sequencer (Life Technologies), with 200-bp read chemistry using a 318 Chip in two consecutive runs. After quality trimming (>q20), a total of 1,484,357 reads (average length, 275 bp) were assembled using MIRA version 3.9.5 (10) and CLC Genomics Workbench version 6.5. Analysis and further joining of the resultant contigs using the CLC Microbial Genome Finishing Module, after aligning them to the genomes of *A. hydrophila* ML09-119 (accession no. CP005966.1) (7) and *A. hydrophila* ATCC 7966^T^ (accession no. CP000462.1) (11), generated 59 consensus contigs (731 to 297,409 nucleotides). *De novo* assembling of all unmapped reads, followed by mapping back to consensus contigs and joining them whenever possible, resulted in a final assembly of 28 contigs (mean size, 168,039 bp; maximum length, 762,403 bp). The total size of the draft genome (4,705,099 nucleotides [nt]) and the G+C content (61.6%) are in good agreement with the respective figures for the published *A. hydrophila* genomes (4.5 to 5.0 Mb and 60.8 to 62%, respectively).

Annotation of the genome using RAST (12) identified 4,256 protein-coding sequences. A total of 117 tRNAs and 31 rRNAs were predicted using tRNAscan-SE 1.23 (13) and RNAmmer 1.2 (14), respectively. RAST predicted numerous genes encoding virulence and defense factors, of which 67 are related to resistance to antibiotics/toxic compounds. These include 20 genes for multidrug resistance efflux pumps, 2 for β-lactamases, 1 multiple-antibiotic resistance locus, 1 gene for a lysozyme inhibitor, and 4 genes encoding fluoroquinolone resistance. PHAST (15) detected two intact prophages (40.5 and 29.5 kb) showing 77% and 69% identities, respectively, to phiO18P, a bacteriophage from *Aeromonas media* (16).

The *A. hydrophila* Ae34 draft genome sequence will provide insights into the specific genomic features responsible for its opportunistic pathogenicity in ornamental fish and will be of use for the development of diagnostic and preventive strategies to combat aeromonad infections in fish.

### Nucleotide sequence accession numbers.

The genome sequence has been deposited in the DDBJ/EMBL/GenBank databases under accession no. BAXY01000001 to BAXY01000028.
